# Obesity, the Endocannabinoid System, and Bias Arising from Pharmaceutical Sponsorship

**DOI:** 10.1371/journal.pone.0005092

**Published:** 2009-03-31

**Authors:** John M. McPartland

**Affiliations:** Department of Osteopathic Manipulative Medicine, Michigan State University, East Lansing, Michigan, United States of America; Copenhagen University Hospital, Denmark

## Abstract

**Background:**

Previous research has shown that academic physicians conflicted by funding from the pharmaceutical industry have corrupted evidence based medicine and helped enlarge the market for drugs. Physicians made pharmaceutical-friendly statements, engaged in disease mongering, and signed biased review articles ghost-authored by corporate employees. This paper tested the hypothesis that bias affects review articles regarding rimonabant, an anti-obesity drug that blocks the central cannabinoid receptor.

**Methods/Principal Findings:**

A MEDLINE search was performed for rimonabant review articles, limited to articles authored by USA physicians who served as consultants for the company that manufactures rimonabant. Extracted articles were examined for industry-friendly bias, identified by three methods: analysis with a validated instrument for monitoring bias in continuing medical education (CME); analysis for bias defined as statements that ran contrary to external evidence; and a tally of misrepresentations about the endocannabinoid system. Eight review articles were identified, but only three disclosed authors' financial conflicts of interest, despite easily accessible information to the contrary. The Takhar CME bias instrument demonstrated statistically significant bias in all the review articles. Biased statements that were nearly identical reappeared in the articles, including disease mongering, exaggerating rimonabant's efficacy and safety, lack of criticisms regarding rimonabant clinical trials, and speculations about surrogate markers stated as facts. Distinctive and identical misrepresentations regarding the endocannabinoid system also reappeared in articles by different authors.

**Conclusions:**

The findings are characteristic of bias that arises from financial conflicts of interest, and suggestive of ghostwriting by a common author. Resolutions for this scenario are proposed.

## Introduction

The epidemic of obesity began, as many modern epidemics do, with a reclassification. In 1998, the number of overweight and obese individuals in the USA swelled instantaneously by 37 million, when a NIH task force redefined overweight as a body mass index (BMI) ≥25 kg/m^2^, and obesity as BMI ≥30 [1]. The task force was criticized for ignoring studies that disputed BMI as a valid surrogate marker for adiposity, and for circuitously basing its reclassification upon opinions and flawed studies authored by its own members, rather than independent studies [2], [3]. Nearly 90% of the obesity task force members had financial ties to the weight-loss industry, including pharmaceutical companies and weight loss clinics [4]. The Chair of the task force stated that pharmaceutical corporations “have no influence over what I say. … I'm not accepting payment directly. It comes through a company that runs continuing education. Maybe that's a bad thing. But if you did away with this, you would wipe out 80 percent of the medical education programs” [4]. The Chair of the task force was former President of the NAASO (North American Association for the Study of Obesity, also called the Obesity Society). The NAASO is an accredited continuing medical education (CME) provider. The ex-President approved NAASO CME programs as free of commercial bias [5], [6], although the programs were funded by Sanofi-Aventis (the manufacturer of a weight-loss drug), and he received financial support from Sanofi-Aventis [7]. A year after chairing the obesity task force, the ex-President was identified in a lawsuit as the guest author of a ghostwritten review on obesity commissioned by Wyeth-Ayerst regarding long-term, off-label use of “fen-phen” (fenfluramine and phentermine) [8].

In 2004, researchers from the Centers for Disease Control (CDC) reported that obesity caused 400,000 deaths in the year 2000 [9]. Despite the fact that this statistic was unsupportable (and was downsized after a congressional inquiry [10]), Medicare officials promptly announced they would treat obesity as a disease, opening the way for government reimbursement of treatments [11]. A second group of researchers reanalyzed the data used to generate the 400,000 number. After adjusting for confounding factors, obesity-related deaths in 2000 numbered 25,814 – less than 7% of the original estimate [12]. U.S. Surgeon General Richard Carmona subsequently announced, “Obesity is the terror within…the magnitude of the dilemma will dwarf 9–11 or any other terrorist attempt” [13]. Severe obesity is an indisputable health hazard, and its prevalence is rising. But the framing of obesity as a 9–11 terror is an example of *disease mongering,* which includes the promotion of new diseases, the expansion of illness boundaries, the medicalization of normal physiology, and the expansion of markets for disease treatments [14].

### Cannabinoids, evidence based medicine, and surrogate markers

Two new anti-obesity drugs, rimonabant (Acomplia®, Sanofi-Aventis) and taranabant (Merck), work by a new mechanism: blockade of the cannabinoid 1 (CB_1_) receptor, an integral part of the endocannabinoid system (ECS) [15]. Obesity leads to excessive endocannabinoid production, which drives CB_1_ in a feed-forward dysfunction [16]. Endocannabinoids as well as plant cannabinoids in marijuana stimulate appetite, so it makes sense that a CB_1_ antagonist would suppress appetite. However, endocannabinoids do more than modulate appetite. The ECS plays important roles in neurogenesis, neurodegenerative diseases, mood disorders, pain perception, gut function, immunity, and inflammation [17]. These important roles suggest that ECS blockade might cause adverse effects. However, this type of physiological rationale is not accepted by evidence based medicine (EBM) guidelines [18]. EBM accepts randomized clinical trials (RCTs) as best evidence. Pharmaceutical corporations increasingly recognize the value of RCTs in shaping EBM. They treat RCTs as important resources to be managed, thereby extending their marketing arm into the peer-reviewed medical literature [19]. Pharmaceutical corporations spent US$57.5 billion *on marketing alone* in 2004. This was substantially greater than US$31.5 billion expended on domestic pharmaceutical research [20].

Four rimonabant-in-obesity (RIO) RCTs, all funded and conducted by Sanofi-Aventis, have been published, although 25 RCTs of rimonabant for the treatment of obesity and diabetes are completed or underway [21]. This 4-to-25 ratio suggests “publication bias,” which arises when pharmaceutical corporations choose not to publish unfavorable studies [22], [23]. Criticisms of the RIO trials included the use of unvalidated or disputed surrogate endpoints [24], [25], favorable claims not supported by trial data [26], overstated treatment efficacy [27], downplayed adverse effects [25], [28], [29], lack of internal validity and external validity or generalizability [30], [31], and failure to disclose financially-conflicted interests [32]. However, these criticisms and other types of narrative reviews are not recognized by EBM; EBM relies upon meta-analyses of RCTs [18]. A meta-analysis of the RIO RCTs concluded that rimonabant was safe and effective [33]. The meta-analysis was funded by Sanofi-Aventis. Industry-funded meta-analyses tend to be less transparent, have more methodological flaws, and make more pro-industry conclusions regarding drugs than do independent meta-analyses [34]. Consistent with this, four independent meta-analyses of the RIO trials have questioned rimonabant's efficacy and potential for adverse effects [26], [35]–[37].

One taranabant clinical trial has been published in the peer-reviewed literature [38]. The study also used unvalidated or disputed surrogate endpoints, made claims not supported by trial data, and downplayed adverse effects. The use of surrogate endpoints instead of clinical end points has come under recent scrutiny. Surrogate markers help get drugs to the market quickly, but they may not correlate with disease outcome. Just because patients with flu have a fever, for example, doesn't mean that treating the fever will clear the infection [39]. Rosiglitazone was fast-tracked through FDA approval because it lowered serum glucose levels, but a meta-analysis showed that rosiglitazone increased the risk of myocardial infarction [40]. According to Steven Nissen, author of the meta-analysis, “Wait long enough, and you're going to find that all surrogates eventually fail due to these off-target effects” [39]. Serum cholesterol serves as a surrogate for cardiovascular disease, and ezetimibe-simvastatin lowers serum cholesterol, but ezetimibe-simvastatin did not slow the development of atherosclerosis in patients [41]. Rimonabant and taranabant RCTs employed several disputed surrogate markers. Even “metabolic syndrome,” a composite surrogate measure, has limited value as a cardiovascular risk marker [42], [43]. Sanofi-Aventis answered these criticisms with the *STRADIVARIUS* study, which measured rimonabant's effects upon coronary artery atheroma volume [44]. The study was conducted by Steve Nissen, a champion of clinically-relevant outcome measures [39], [40], [45]. Coronary artery atheroma volume, however, is a nonvalidated surrogate endpoint for cardiovascular outcomes [46], [47]. The study showed that rimonabant had no effect on percent atheroma volume, although Nissen and colleagues noted improvements in *secondary* nonvalidated surrogate endpoints, such as normalized total atheroma volume [44].

### ECB, CME, and review articles

In addition to RCTs and meta-analyses, clinicians base rational EBM decisions upon CME presented by fellow physicians. Clinicians must participate in CME to fulfill licensure requirements, making them a “captured audience” for corporate-sponsored messages. Pharmaceutical corporations routinely seed CME with review articles that promote their products, thereby further unraveling EBM [48]. Review articles often contain industry bias [49], [50], especially articles in journal supplements, which are not usually peer reviewed [19]. Journal supplements are quite lucrative to medical journals, because pharmaceutical corporations sponsor them. Corporate employees may ghostwrite review articles, and then influential physicians are recruited to sign the articles [51]. Whereas authorship establishes accountability and responsibility, ghost authorship increases the potential for conflicted manipulation. Documents made public in litigation showed that Wyeth-Ayerst [8], Pfizer [52], [53], and Merck [54] employed corporate authors to ghost author CME review articles.

In the past few years, several CME review articles of rimonabant have been published. Some authors of the review articles also served on the NIH obesity task force, the NAASO board, and coauthored RIO publications. One rimonabant review article was presumably ghostwritten because the authors were listed as “editors,” without identifying a primary author, and a Sanofi-Aventis copyright appeared in small print on the back cover of the supplement [55]. The purpose of this paper was to test the hypothesis that rimonabant review articles expressed a high incidence of bias.

## Methods

Review articles were identified through a MEDLINE search using the keywords endocannabinoid AND obesity AND rimonabant, limited to articles published prior to 2007 (when the FDA reviewed rimonabant). To be included in the analysis, a review article had to meet the following criteria:

- ample information (≥2 paragraphs) describing the ECS system and obesity;
- ample information (≥1 paragraph) describing obesity and rimonabant;
- authored by a USA physician who received financial support from Sanofi-Aventis.

Because the study aimed to uncover identical bias by different authors, only one article by each author was analyzed; the earliest published article was analyzed and subsequent publications were excluded. Evidence of real or potential conflicts of interest (i.e., financial support in the form of honoraria, education support, research funding, or identification as a consultant or speaker) was obtained by searching Google using each physician's name combined with Sanofi-Aventis. Biases and misrepresentations were measured by three methods:

- Review articles were analyzed with a validated instrument for monitoring bias in CME [56]. The Takhar instrument consisted of 13 questions (see Table 1), with scores graded on a scale from 1 (strongly disagree this paper displays bias) to 4 (strongly agree this paper displays bias), or N/A (not applicable). The instrument was designed for oral CME presentations, so it was slightly modified (e.g., “author” instead of “speaker”).
- The “RIO bias tally” searched articles for biased statements or inappropriate omissions that originally appeared in the RIO publications (see Table 2), graded on a scale from 0 (no bias evident in review article), 1 (a mix of biased and unbiased statements) to 2 (bias evident), or N/A (no statement made regarding the item).
- Articles were scanned for recurrent themes and for identical misrepresentations about the ECS written by different authors.

**Table 1:** Thirteen questions (A to M) comprising the Takhar instrument [56] for monitoring bias in Continuing Medical Education (*with adaptations applied to rimonabant review articles in italics*)

A.	Conflict of interest was *not* declared by the author with a disclosure statement.
B.	Commercial interest was clearly present (*via the Sanofi name or company logo, product branding, illustrations reproduced or adapted from Sanofi publications, or reference to a medical education and communication company*).
C.	Valid, credible evaluation of peer-reviewed evidence-based medicine (EBM) was *not* used in the presentation, based on my perception.
D.	The author did *not* integrate his or her clinical expertise with the best available EBM in his/her presentation.
E.	The data were presented in an unbalanced manner, and some outcomes were favored over others (i.e., data were presented that favored one company's products over another's).
F.	Published sources were identified for evidence reported.
G.	The data presented in the program were incomplete or framed in a biased fashion.
H.	Rival drugs for treatment of obesity were not mentioned (*e.g., orlistat and sibutramine, or if mentioned, their adverse affects were emphasized over their efficacy*).
I.	Trade names of the drug were used (*Acomplia or rimonabant, named after the Sanofi lead researcher, * ***RI*** *naldi-Car* ***mona***), rather than generic names (*SR141716 or SR141716A*).
J.	If unapproved uses of drugs were discussed, the author informed the audience of this according to current guidelines.
K.	The paper does not contribute to the best interests of patients.
L.	The paper promotes marketing of drug knowledge.
M.	This program enhances medical knowledge.

**Table 2:** Industry-friendly biased statements (or biased omissions) that appeared in at least seven out of eight rimonabant review articles (*with evidence contrary in italics*)

1.	Disease mongering: the ECS requires pharmacological blockade because it induces detrimental effects: overfeeding, obesity, diabetes, hyperlipidemia, and/or hepatic steatosis. *Review articles did not mention the many beneficial effects of the ECS, which include anti-inflammatory and analgesic effects, immunomodulatory and neuroprotective effects, and beneficial mood-altering effects * *[17]* *.*
2.	Weight reduction from rimonabant was described as “appreciable,” ”large,” “dramatic,” etc. *Weight loss was modest: less than 5% of total body weight. Trial participants with a mean weight of 99.6 kg (219 lb) on the highest dose of rimonabant lost 4.7 kg (10.4 lb) compared to placebo * *[26], [35]–[37]* *. Participants re-randomized to placebo regained most of their weight.*
3.	Rimonabant's reduction of high-density lipoprotein (HDL) cholesterol was highlighted, while no mention was made of its inability to lower total cholesterol or LDL. *Rimonabant produced a statistically significant—but clinically marginal—3.5 mg/dl increase in HDL. No improvements were seen in total cholesterol or LDL cholesterol * *[26], [35]–[37]* *.*
4.	Adverse effects were not mentioned or were described as “mild,” “transient,” “well tolerated,” or “slightly greater than placebo.” *Rimonabant caused significantly more adverse events than did placebo; trial participants given rimonabant were 2.5 times more likely to discontinue the treatment because of depressive mood disorders than were those given placebo * *[37]* *.*
5.	The external validity (or generalizability) of the RIO trials went unquestioned. *Potential trial participants with depression were excluded from RIO trials; in actual clinical practice about half of patients seeking treatment are depressed * *[30], [37]* *.*
6.	Methodological weaknesses (internal validity) in RIO trials went unmentioned; or if high drop-out rates were mentioned, they were justified as “typical of obesity trials.” *The authors of two RIO trials did not report appropriate methods of randomization or allocation concealment, and none provided details regarding blinding of participants or treatment providers * *[35]* *. High dropout rates and nonadherence in all RIO trials may have resulted in overestimation of the benefits of treatment * *[29]* *.*
7.	Relative contraindications other than depression were not mentioned. *The rimonabant package insert advises against taking rimonabant when breast-feeding or pregnant, advises “special care” in patients with impaired liver or renal function, epilepsy, or who are under 18 years of age, and warns against co-administration with cytochrome P-450 CYP3A4-modulating drugs * *[87], [88]* *.*
8.	Inappropriate surrogate markers went unquestioned. BMI of ≥25 was used as a surrogate marker for adiposity and an accurate predictor of mortality. *BMI is not a good proxy for adiposity; BMI fails to account for age, gender, ethnicity, fat distribution, physical conditioning, and disease state * *[3]* *. Mortality may not increase significantly until BMI >35, and mortality may actually be lowest in the BMI 25–30 range * *[12], [89]* *.*
9.	Competing drugs were not mentioned or mentioned only in a way that highlighted adverse effects. *No evidence supports the superiority of rimonabant to orlistat and sibutramine: no head-to-head comparisons have been done.*
10.	No mention was made of rimonabant potentially counteracting drugs or other therapeutic interventions that augment the ECS. *Endocannabinoid tone or CB_1_ expression are enhanced by paracetamol (acetaminophen), nonsteroidal anti-inflammatory drugs, tricyclic antidepressants, diazepam, dexamethasone, and docosahexaenoic acid (fish oil) supplements, as well as aerobic exercise, spinal manipulation, massage, and perhaps acupuncture * *[15]* *.*

## Results

Eight review articles were identified that met inclusion criteria [57]–[64]. Only three of eight articles disclosed authors' conflicts of interest with Sanofi-Aventis. Two articles carried the statement that the author had “*no conflict of interest,”* despite easily accessible information to the contrary—searches with Google revealed all the authors served as consultants, or on speaker bureaus, or received other financial support from Sanofi-Aventis.

The Takhar CME bias instrument demonstrated bias in all the review articles (Table 3), with mean scores ranging from 2.6 (weakly biased) to 3.6 (strongly biased). Collectively, the mean Takhar score was 3.08 (95% CI: 2.78 to 3.39). Ten industry-friendly statements that originally appeared in RIO publications reappeared in the eight review articles (Table 3). Biased statements included disease mongering, speculations regarding surrogate markers stated as facts, lack of acknowledgment of RIO design flaws, and exaggerated statements of rimonabant's efficacy and safety. Mean scores from the Takhar bias instrument (Table 3) correlated with mean scores from the RIO bias tally (Table 3), but fell short of statistical significance (*r* = 0.50, *p* = 0.11).

**Table 3:** Bias in eight rimonabant review articles.^1^

Takhar bias scale item	Eight review articles (citation numbers from Reference section)								RIO bias tally item
	[57]	[58]	[59]	[60]	[61]	[62]	[63]	[64]	
A	4 / 1	3 / 2	4 / 2	1 / 2	4 / 2	4 / 2	4 / 2	1 / 2	1
B	3 / 2	3 / 2	3 / 2	4 / 2	3 / 2	3 / 2	4 / 2	4 / 2	2
C	3 / N/A	3 / 2	3 / 2	3 / 2	3 / 2	3 / 0	3 / 2	3 / 2	3
D	3 / 2	2 / 1	3 / 1	4 / 2	2 / 0	1 / 1	4 / 0	3 / 1	4
E	4 / 2	2 / 2	3 / 2	4 / 2	3 / 2	2 / 2	4 / 2	3 / 2	5
F	2 / 2	2 / 2	2 / 2	3 / 2	2 / 2	2 / 2	2 / 2	2 / 2	6
G	4 / 2	3 / 2	3 / 2	4 / 2	4 / 2	4 / 2	4 / 2	4 / 2	7
H	4 / 1	2 / 2	2 / 2	4 / 2	4 / 2	2 / 2	4 / 2	3 / 2	8
I	3 / 2	3 / 1	3 / 0	3 / 2	3 / 2	3 / 0	3 / 2	3 / 1	9
J	N/A	N/A	N/A	N/A	N/A	N/A	N/A	N/A	10
	/ 2	/ 2	/ 2	/ 2	/ 2	/ 2	/ 2	/ 2	
K	2	2	2	3	2	2	3	2	
L	2	3	4	4	3	4	4	4	
M	2	3	3	4	3	3	4	3	
Takhar mean	3.0	2.6	2.9	3.4	3.0	2.8	3.6	2.9	
	1.78	1.8	1.7	2.0	1.8	1.5	1.8	1.8	RIO bias mean

1 Each article was scored with the Takhar bias instrument (items A to M in the first column, see Methods and Table 1), followed by a back-slash (/), and then scored with the RIO bias tally (items 1 to 10 in the last column, see Methods and Table 2)

Recurrent visual and textual themes emerged from the eight review articles. Graphics from Sanofi-Aventis promotional materials [55] reappeared in a review article [61]. Articles by different authors in different journals nevertheless used similar stock photos from Getty Images, Inc. (e.g., [60] and [63]). Three unusual yet identical misrepresentations about the ECS appeared in articles by different authors:

1. **The hypothalamus was named** ***first*** **in descriptions of CB_1_ expression in the brain**. Three of eight articles shared this misrepresentation [57], [58], [63]. Actually CB_1_ expression is relatively low in the hypothalamus. In human brain, the rank order of CB_1_ receptor density is: substantia nigra>globus pallidus>hippocampus>cerebral cortex>putamen>caudate>cerebellum>amygdala>thalamus = hypothalamus [65]. This error may arise from the fact that Sanofi-sponsored research has focused upon the hypothalamus (e.g., [66]).
2. **Adipose tissue was listed amongst tissues with dense CB_1_ expression.** Six of eight articles stated this [57]–[61], [63]. Sanofi-sponsored research has highlighted adipose tissue in rimonabant's “peripheral effects” (e.g., [67]). However, most independent studies have found that CB_1_ expression is relatively low or undetectable in adipose and adipocyte-rich tissue such as bone marrow [68]–[71]. Adipocyte CB_1_ expression actually *decreases* in obese research participants [67], [72].
3. **Hepatic tissue was listed amongst tissues with dense CB_1_ expression.** Five of eight articles stated this [57], . Sanofi-sponsored researchers claim that hepatocytes contribute to rimonabant's peripheral effects in mice [73]. On the other hand, CB_1_ expression is sufficiently low that some independent studies failed to identify CB_1_ in liver at all [68], [70]. Recent studies have detected CB_1_ in fibrotic liver cells; hepatic CB_1_ expression in humans may be limited to cirrhotic or other pathological conditions [74].

## Discussion

All the authors of rimonabant review articles held academic positions, many at prestigious institutions. They typified “medical opinion leaders” sought by pharmaceutical corporations to sign ghostwritten articles [48], [52], [53], [75]. Seven of eight rimonabant review articles appeared in journal supplements, which are non-peer-reviewed, usually industry-funded publications known to carry a high incidence of bias [19]. The Takhar bias instrument demonstrated bias in all eight articles (Table 3). The mean score was 3.08 (95% CI: 2.78 to 3.39), significantly greater than the 2.5 score that separates unbiased from biased publications [56]. In comparison, the mean score for 17 accredited CME events evaluated by Takhar and colleagues was 1.65 (95% CI: 1.32 to 1.99) [56]. An additional analysis of non-Sanofi-supported review articles would provide compelling data for comparison. But “absence of evidence is not evidence of absence”—to prove lack of Sanofi support would require much more than a Google search.

The RIO bias tally identified ten nearly-identical industry-friendly statements or inappropriate omissions in articles written by different authors (Table 3). These statements originally appeared in RIO publications, which acknowledged editorial assistance by Sanofi-Aventis (e.g., [7]), whereas the review articles did not. Nearly identical illustrations reappeared in several articles, and distinctive factual misrepresentations reappeared in articles by different authors. Replication of passages in a single author's work may indicate only carelessness, but replication of passages in articles by different authors raises the question of whether a common ghost author was involved. One rimonabant review article (not included in the analysis because we found no evidence that Sanofi funded its authors) listed the authors as “editors,” and the primary (ghost-) author was unidentified [55]. Of course, judgment regarding ghostwriting or plagiarism should be withheld until the candidate publications are appraised by an editorial board or ethics committee. The same proviso was made by Errami and Garner [76], who used a computational text-searching algorithm to identify “duplicate publications” and “plagiarism” in abstracts cited by PubMed. A search of their database (http://spore.swmed.edu/dejavu/) using the keyword “rimonabant” revealed seven pairs of duplicate publications (including one review article identified herein). The cases of suspected plagiarism repeatedly engaged in disease mongering, which expands the market for those who sell disease remedies. Disease mongering and “supersizing” of rimonabant's indications have been criticized [43], and satirized by a description of “indolebant,” a fictional CB_1_ antagonist that treats “extreme laziness” [77].

Rationally choosing the best medication, like other sorts of clinical decision-making, has increasingly relied upon EBM. Thus whoever generates EBM, by funding RCTs, meta-analyses, and CME, may bias clinical decision-making regarding pharmaceuticals [78]. Industry-friendly bias is not unique to rimonabant publications. The discovery process in recent litigation has revealed that many pharmaceutical corporations recruit and train influential physicians for the purpose of manipulating their colleagues. These physicians sign biased ghostwritten articles without disclosing conflicts of interest [48], [52], [53], [75]. For participating in Parke-Davis promotional CME efforts, physicians received honoraria up to US$158,250, in addition to paid travel, lodging, and amenities at luxury resorts [53]. This behavior is not limited to MDs — a pharmaceutical marketing disclosure law in Vermont revealed that the third highest recipient in the state was an osteopathic physician who received $99,843 in 2007 [79]. Despite the fact that osteopathy began as an essentially drug-free school of medicine, the pharmaceutical industry now imparts significant financial leverage over that profession.

Financial conflicts of interest also bias clinical practice guidelines and FDA decisions. An analysis of 44 clinical guidelines revealed 87% of panelists received financial support from pharmaceutical companies, yet only two of the guidelines disclosed panelists' financial conflicts [80]. A larger investigation of over 200 guidelines revealed about 70% of guideline panels being affected [81]. In one guideline panel, every panel member was paid by a drug manufacturer, and that manufacturer's drug was recommended by the panel [81]. Members of FDA drug advisory committees have financial conflicts but rarely recluse themselves from voting, and they tend to vote in favor of corporations that sponsored them [82]. Ten of the 32 FDA panelists that voted in favor of rofecoxib and valdecoxib received fees from the makers of those drugs [83]. Had those financially conflicted panelists been reclused, the FDA would have voted against continued sales of rofecoxib and valdecoxib [83].

In summary, financial conflicts permeate the system and are by no means limited to corporations referenced in this article, such as Merck, Parke-Davis, Pfizer, Sanofi-Aventis, and Wyeth-Ayerst. On balance, pharmaceutical corporations do good work and aid in humanitarian efforts. For example Sanofi-Aventis provides artemisinin at cost to malaria-endemic countries [84]. Nevertheless, ghost authorship and the corrupting effects of covert financial support must cease. Only three of eight rimonabant review articles disclosed corporate sponsorship; two authors specifically denied conflicts. Lack of disclosure prevents readers from judging the credibility of an author. Medical journals should require stronger author disclosure procedures, and universities should discipline academics who sign ghostwritten articles. This behavior should be regarded as unethical misconduct [85]. More broadly, researchers with conflicts of interest should not be allowed to sit on guideline committees and regulatory boards. Corporate funding of CME programs and review articles should be abolished.

### Post script

While this paper was under review, Merck halted taranabant RCTs, and Sanofi-Aventis removed rimonabant from the European market. The FDA rejected rimonabant after data submitted by Sanofi-Aventis revealed adverse effects in RIO trials that went unreported in RIO publications [86], including one death in a rimonabant-treated subject (ruled a suicide by the FDA, [86]) that did not appear in the pertinent publication [7]. Although the risk-benefit ratio of cannabinoid receptor blockade may preclude its use for *chronic* conditions such as obesity and drug or alcohol dependence, cannabinoid receptor blockade could serve in the treatment of *acute* endocannabinoid dysregulation, such as hepatic cirrhosis, hemorrhagic or endotoxic shock, cardiac reperfusion injury, and doxorubicin-induced cardiotoxicity [15].
